# Women Using Mobile Phones for Health Communication Are More Likely to Use Prenatal and Postnatal Services in Bangladesh: Cross-Sectional Study

**DOI:** 10.2196/10645

**Published:** 2019-02-28

**Authors:** Shangfeng Tang, Bishwajit Ghose, Md Rakibul Hoque, Gang Hao, Sanni Yaya

**Affiliations:** 1 School of Medicine and Health Management, Tongji Medical College, Huazhong University of Science and Technology Wuhan China; 2 School of International Development and Global Studies, University of Ottawa Ottawa, ON Canada; 3 Department of Management Information Systems, Faculty of Business Studies, University of Dhaka Dhaka Bangladesh; 4 Zunyi Medical and Pharmaceutical College Guizhou China

**Keywords:** antenatal care, facility delivery services, postnatal care, mHealth, Bangladesh

## Abstract

**Background:**

The public health system in Bangladesh has been struggling to provide coverage and utilization of basic maternal health care services in pursuit of achieving maternal and child mortality-related goals. Interestingly, the rapid popularization of mobile technology in the country is transforming the landscape of health care access and delivery. However, little is known regarding the use of mobile phones from the perspective of maternal health care service utilization.

**Objective:**

In this study, we aimed to investigate the prevalence and sociodemographic pattern of mobile phone use for health services among women and relationship between the use of mobile phone use and the uptake of essential maternal health services (MHSs).

**Methods:**

Cross-sectional data from the Bangladesh Demographic and Health Survey on 4494 mothers aged between 15 and 39 years were used in the analysis. Using mobile phones to get health services or advice was hypothesized to have a positive association with the uptake of basic MHSs (antenatal care, ANC, facility delivery services, postnatal care) and postnatal care for the newborn. Data were analyzed using bivariate and multivariable techniques.

**Results:**

More than a quarter (1276/4494, 28.4%; 95% CI 26.8-30.3) of the women aged 15-39 years reported using mobile phones to get health services with significant sociodemographic variations in the use of mobile phones. Analysis of the specific purposes revealed that, in most cases, mobile phones were used to contact service providers and consult with the same about what to do, whereas a smaller proportion reported using mobile phone for the purposes of arranging money and transportation. Multivariable analysis showed that compared with respondents who reported not using mobile phones for health care services, those who used them had higher odds of making 3+ ANC visits and delivering at a health facility. The odds were slightly higher for rural residents than for those in the urban areas.

**Conclusions:**

The findings of this study conclude that women who use mobile phones are more likely to use ANC and professional delivery services than those who do not. More in-depth studies are necessary to understand the mechanism through which mobile phone-based services enhance the uptake of maternal health care.

## Introduction

The demography of Bangladesh is usually characterized by total fertility rate and maternal and child mortality [1,2]. Like all other south Asian nations, widespread poverty, poor health care infrastructure, inequality in access to care, and low health literacy constitute some of the major causes of underutilization of essential maternal health care services (eg, antenatal care, ANC, and professional services for childbirth) that translate to higher burden of maternal and child mortality, which is recognized as a serious public health problem in the country [1,3,4]. Over the past few decades, the Ministry of Health and Family Welfare (MoHFW) has developed and implemented strategies for tackling high maternal and child mortality rates through programmatic intervention within national (Population and Health Policy), international (Millennium Development Goal, 5) [5], and joint (Maternal and Neonatal Health Initiatives in Bangladesh) [6] policy frameworks. Millennium Development Goals progress reports reveal significant gains in terms of decline in both maternal and child mortality rates with an estimated reduction of 57% in child mortality and 66% in maternal mortality [1]. The statistics on essential maternal health service uptake (Millennium Development Goal 5) on the other hand appear to be less encouraging because the figures remain well behind the internationally agreed targets: increase in facility delivery between 2001 (9%) and 2010 (23%) and a 25% increase in ANC uptake between 1996-1997 (29%) and 2010 (54%) [7]. Despite the growing public and private sector initiatives to promote the utilization of maternal health services (MHSs), progress is slackened by persistent issues, for example, the lack of human resources in health care, high out-of-pocket expenditure, and inequitable access to quality services, especially among the marginalized and rural population [1].

Amid this multitude of challenges, the hope is that the wide availability and progressive expansion of mobile and internet technology could open windows for more expansive and equitable care delivery to communities otherwise deprived from accessing basic health care. It is no longer a new concept that telemedicine or the application of information and communication technology in the public health sector can substantially improve the quality of care and efficiency in administrative and managerial tasks in a cost-effective manner [8-10]. In developed countries, telemedicine has so far earned massive popularity as a convenient, cost-effective, and time-saving platform for almost all aspects of preventive, curative, and rehabilitative care including obstetrics and gynecology, psychiatry, and self-management of chronic noncommunicable diseases. In contrast, mobile health (mHealth) technology is still in its infancy in Bangladesh and other south Asian nations but has been gaining growing attention among health and policy experts. Evidence suggests that mHealth is highly effective in reducing financial and transportation barriers and facilitates basic MHSs as well as emergency care during emergency obstetric referrals in low-income settings [11,12]. Therefore, mHealth is increasingly seen as a key strategy to promoting maternal and child health in the developing countries [13-16].

So far, countries in south Asia, especially India and Bangladesh, have embarked on various electronic health (eHealth) initiatives (eg, electronic health records at the managerial level) and provision mHealth services as a means to ameliorate primary health care services at the population level [17,18]. Although the first eHealth initiative was taken by MoHFW in 1998, since then, the adoption of this technology has been proliferating mainly in the private sector where the common services include teleconsultation, prescription, and referral [19]. One of the pioneering private eHealth institution in Bangladesh called Telemedicine Reference Center Limited was established in 1999 with the goal of using mobile phones for health care delivery [20]. Services including computerization of health facilities with internet servers and mHealth service for communicating with health care providers were introduced in 2008 [17]. Mobile phone-based initiative for maternal and child care is more recent and was launched by Mobile Alliance for Maternal Action (locally known as Aponjon) in collaboration with MoHFW [21]. Service includes biweekly short message service (SMS) text messages or voice messages from the beginning of conception through the first birthday of the child on vital health information targeted at expectant and new mothers and their relatives [21]. The project became functional in 2012 with the vision of addressing the dire situation of maternal and child health services in the country.

Despite the emerging interests in this field, there is a noticeable absence of large-scale research studies that are necessary to generate the evidence base for informing investors in preventing the initiation of major pilot projects. In a previous study, we have shown the regional disparities among urban women in using mobile phone services for delivery services [1]. In this study, we aimed to investigate what percentage of women are using mobile phones for health services and whether seeking care through mobile phones has any relationship with their utilization of essential maternal and child health care services namely antenatal, facility delivery, and postnatal care for the mother and the newborn.

## Methods

### Survey and Data Source

**Figure 1:**
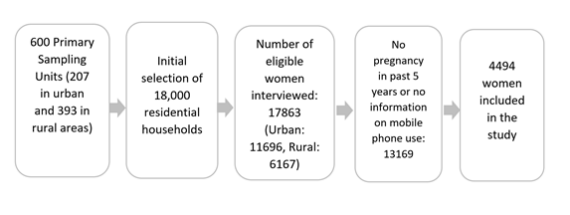
Flowchart of the sampling procedure.

We obtained the data for this study from the seventh round of Bangladesh Demographic and Health Survey (BDHS) 2014. This survey was implemented through a collaborative effort of the National Institute of Population Research and Training, Inner City Fund International, United States, and Mitra & Associates under the financial auspices of the United States Agency for International Development, Bangladesh. The main objectives of the surveys are to provide quality and nationally representative data on crucial health indicators needed for the monitoring and evaluation of national health programs and thereby assisting in policy making, designing public health programs in the country. BDHS adopted a 2-stage stratified technique for selecting sample households. In the first stage, 600 enumeration areas (EAs; primary sampling units with an average of about 120 households) were selected with probability proportional to the EA size with 207 EAs in urban areas and 393 in rural areas. In the second stage, a systematic sample of 30 households on average was selected per EA. This selection was expected to result in completed interviews with about 18,000 individual women, of whom 17,863 were finally interviewed yielding a response rate of 98%. For this study, we included only those participants who reported having a completed pregnancy in the years preceding the survey (Figure 1). Participants gave informed consent before taking part in the survey. All Demographic and Health Surveys are approved by Inner City Fund international and an Institutional Review Board in the host country to make sure that the protocols are in compliance with the US Department of Health and Human Services regulations for the protection of human subjects. A more detailed version of the methodology is published elsewhere [1,3].

### Variables

The outcomes variables included 3 maternal variables: ANC, place of delivery, postnatal care, and 1 newborn care variable (postnatal care for the baby). These variables are described in Table 1 along with the list of independent variables. The timing of first ANC is an important aspect of MHSs, but it was not available for the 2014 survey.

### Data Analysis

Data analyses were carried out using IBM SPSS Statistics version 24. Before analysis, the dataset was cleaned, tested for multicollinearity, and checked for cases that fulfilled the inclusion criteria, experience of at least 1 childbirth in the preceding 5 years, providing information on mobile phone usage. Owing to the clustered nature of the survey, we used a complex sample analysis technique that takes into account the sampling weight, strata, and clusters. Participants’ demographic characteristics were presented using frequencies and percentages. Pearson chi-square tests were performed to examine the bivariate tests of association between the dependent and explanatory variables. Variables that showed association at *P*<.1 were retained for multivariable analysis. Binary logistic regression analyses were used to calculate the odds ratios of using the 3 types of maternal and postnatal care for newborns. Both crude and adjusted odds ratios were calculated. At this stage, *P* values (two-tailed) were considered statistically significant only when below .05.

**Table 1:** Description of dependent and independent variables.

Variables			Description
**Dependent variables**			
	Antenatal care visits	Number of antenatal care visits during the last pregnancy. It was categorized as adequate (4 and 4+) and inadequate (0-3)	
	Place of delivery	Place where respondents delivered the most. It was categorized as Home (home of the respondent or relatives) and Heath facility (eg, hospitals, clinics, maternal health centers)	
	Postnatal checkup for mother	Mother’s health checkup by a professional after delivery (Yes or No)	
	Postnatal checkup for baby	Baby’s health checkup by a professional after delivery (Yes or No)	
**Independent variables**			
	Age	Age of the respondent. The survey year was categorized into 5-year groups: 15-19, 20-24, 25-29, 30-34, and 35-39.	
	Division	Seven administrative regions: Barisal, Chittagong, Dhaka, Khulna, Rajshahi, Rangpur, and Sylhet	
	Residency	Type of place of current residence: Urban or Rural	
	Education	Educational attainment based on the number of years of formal schooling: Nil, Primary, Secondary, or Higher	
	Religion	Islam, Hinduism, or others	
	Wealth index	Calculated based on household possession of durable goods (eg, TV^a^, radio, bicycle) and housing quality (eg, type of floor, wall, and roof). Each item is assigned a factor score generated through principal component analysis, which is then summed and standardized for the households. These standardized scores place the households in a continuous scale based on relative wealth scores. The scores were thus obtained from a continuous scale and subsequently categorized into quintiles to rank the household as Poorest, Poorer, Middle, Richer, or Richest.	
	Employed	Employment status of the respondent: Yes or No	
	Reads newspaper	Frequency of reading newspaper: weekly or less than weekly: yes, and do not read at all: No	
	Uses TV or radio	Frequency of using TV or radio: weekly or less than weekly (Yes) and do not use at all (No)	

^a^TV: television.

## Results

The analysis included 4494 women aged 15 to 39 years. The basic sociodemographic profile of the participants is presented in Table 2. The prevalence of attending 4+ ANC visits was 32.0% (1440/4494; 95% CI 29.5-33.2), facility delivery 40.1% (1801/4494; 95% CI 38.3-42.1), and postnatal checkup for the mother 65.7% (2953/4494; 95% CI 64.0-67.7) and for the baby 64.9% (2917/4494; 95% CI 64.0-67.6; not shown in the table). More than a quarter (1276/4494, 28.4%; 95% CI 26.8-30.3) of the women reported using mobile phones to get health services. Those who reported ever using a mobile phone to get health services had significantly higher (*P*=.01) percentages of making at least 4 ANC visits or higher (2543/4494, 56.6%; 95% CI 53.0-60.1) and delivering at a health facility (2435/4494, 54.2%; 95% CI 51.8-58.5). Percentages were also higher for postnatal checkup among mothers and babies but were not statistically significant.

**Figure 2:**
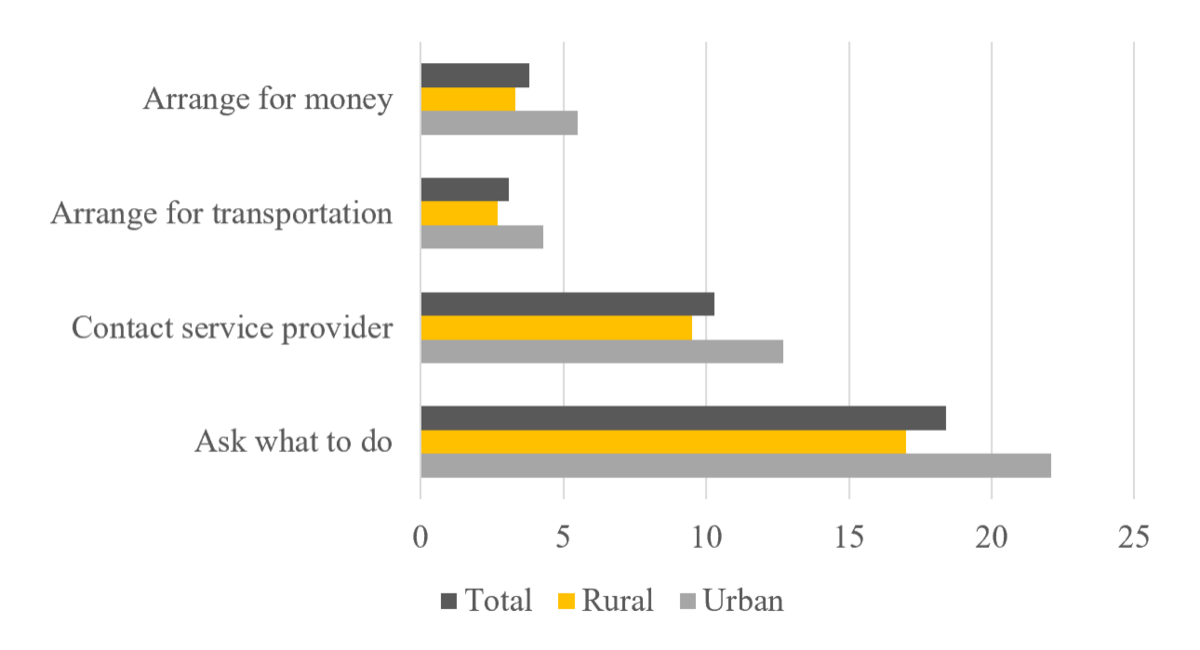
Self-reported purposes (%) for using mobile phone among participants. BDHS 2014.

Respondents were further asked to describe the specific purposes for using a mobile phone. As shown in Figure 2, in most cases, they used mobile phones to contact service providers and consult with the same about what steps to take, whereas a smaller proportion reported on the use of mobile phone for the purposes of arranging money and transportation. The percentage of use varied slightly between urban and rural residents; rural residents made phone calls more frequently than their urban counterparts.

Results of multivariate analysis on the association between the use of mobile phone and uptake of essential MHSs and postnatal care for newborn are presented in Table 3. Results indicate that compared with women who reported not using mobile phones for health care services, those who used had higher odds (adjusted) of achieving an adequate number (4 and 4+) of ANC visits (adjusted odds ratio, AOR 1.612, 95% CI 1.309-1.985) in both urban and rural areas. The odds were slightly higher for rural residents (AOR 1.700, 95% CI 1.317-2.194) than those in the urban areas (AOR 1.454, 95% CI 1.034-2.045). The same was true for delivering at the health facility. Users of mobile phone for health services in rural areas and urban areas had 1.7 times and 1.5 times higher odds, respectively, of delivering at a health facility.

**Table 2:** Sociodemographic profile of the participants (N=4494). Bangladesh Demographic and Health Survey 2014. All the percentages are weighted.

Variables		Participants (N=4494), n (%)	Ever used a mobile phone to get health services or advice				*P* value
			Yes		No		
			%	95% CI	%	95% CI	
**Number of antenatal care visits**							.001
	0-3	3054 (68.00)	43.40	39.90-47.00	73.60	71.30-75.70	
	≥4	1440 (32.00)	56.60	53.0-60.10	26.40	24.30-28.70	
**Place of delivery**							.001
	Home	2693 (59.90)	45.80	42.50-48.20	61.40	58.10-64.70	
	Heath facility	1801 (40.10)	54.20	51.80-58.50	38.60	35.30-41.90	
**Postnatal checkup for mother**							.13
	No	1541 (34.30)	35.70	32.60-39.10	33.50	31.20-35.90	
	Yes	2953 (65.70)	64.30	60.90-67.40	66.50	64.10-68.80	
**Postnatal checkup for baby**							.14
	No	1577 (35.10)	36.50	33.30-39.70	33.30	31.10-35.50	
	Yes	2917 (64.90)	63.50	60.30-66.70	66.70	64.50-68.90	
**Age in years**							.07
	15-19	1531 (34.10)	21.00	18.50-23.70	21.0	19.10-23.00	
	20-24	1147 (25.50)	33.20	30.10-36.60	33.70	31.60-35.90	
	25-29	608 (13.50)	26.90	24.00-29.90	25.20	23.20-27.30	
	30-34	939 (20.90)	14.50	11.50-18.20	13.40	12.10-15.00	
	35-39	269 (60.00)	4.40	3.30-5.80	6.60	5.50-8.00	
**Division**							<.001
	Barisal	532 (11.80)	6.30	4.90-8.00	5.60	4.70-6.60	
	Chittagong	862 (19.20)	26.30	23.20-29.70	20.10	17.90-22.40	
	Dhaka	795 (17.70)	32.50	28.60-36.70	36.40	33.30-39.70	
	Khulna	531 (11.80)	8.30	7.00-9.90	7.90	6.90-9.00	
	Rajshahi	546 (12.10)	9.70	8.10-11.50	10.20	8.90-11.60	
	Rangpur	550 (12.20)	10.10	8.20-12.30	9.60	8.30-11.10	
	Sylhet	678 (15.10)	6.70	5.10-8.80	10.30	8.50-12.30	
**Residency**							<.001
	Urban	1451 (32.30)	31.20	27.90-34.60	24.10	22.00-26.40	
	Rural	3043 (67.70)	68.80	65.40-72.10	75.90	73.60-78.00	
**Education**							<.001
	Nil	607 (13.50)	6.50	5.00-8.40	17.20	15.40-19.20	
	Primary	1235 (27.50)	200	17.30-22.90	31.10	28.90-33.50	
	Secondary	2130 (47.40)	56.60	53.10-60.10	44.10	41.60-46.80	
	Higher	522 (11.60)	16.90	14.50-19.60	7.50	6.50-8.70	
**Religion**							<.001
	Islam	4134 (92.00)	91.40	89.20-93.20	91.80	90.10-93.30	
	Hinduism or others	360 (8.00)	8.60	6.80-10.80	8.20	6.70-9.90	
**Wealth index**							<.001
	Poorest	940 (20.90)	13.70	11.10-16.90	24.80	22.50-27.30	
	Poorer	855 (19.00)	15.00	12.80-17.50	20.50	18.80-22.40	
	Middle	860 (19.10)	19.40	16.90-22.10	18.90	16.80-21.30	
	Richer	946 (21.10)	22.40	19.50-25.70	19.90	18.10-21.90	
	Richest	893 (19.90)	29.50	26.50-32.70	15.80	14.20-17.60	
**Employed**							.01
	No	3511 (78.10)	79.80	76.60-82.70	74.90	72.70-77.10	
	Yes	983 (21.90)	20.20	17.30-23.40	25.10	22.90-27.30	
**Reads newspapers**							<.001
	No	3776 (84.00)	77.00	74.10-79.70	88.50	86.80-90.00	
	Yes	718 (16.00)	23.00	20.30-25.90	11.50	10.00-13.20	
**User of television or radio**							<.001
	No	1796 (40.00)	31.00	27.70-34.50	43.80	41.20-46.30	
	Yes	2698 (60.00)	69.00	65.50-72.30	56.20	53.70-58.80	

**Table 3:** Odds ratios of attending at least 4 antenatal care visits and facility delivery among those who reported using mobile phone for health care services.

Sample group	3+ antenatal care visits		Delivery at the health facility		Postnatal care (mother)		Postnatal care (newborn)	
	COR^a^ (95% CI)	AOR^b^ (95% CI)	COR (95% CI)	AOR (95% CI)	COR (95% CI)	AOR (95% CI)	COR (95% CI)	AOR (95% CI)
Overall	2.032 (1.771-2.567)	1.612 (1.309-1.985)	2.132 (1.771-2.567)	1.612 (1.309-1.985)	0.905 (0.757-1.081)	1.144 (1.001-1.308)	1.134 (0.992-1.295)	0.868 (0.728-1.036)
Urban	1.862 (1.387-2.502)	1.454 (1.034-2.045)	1.862 (1.387-2.502)	1.454 (1.034-2.045)	0822 (0.613-1.104)	0.931 (0.690-1.255)	0.904 (0.671-1.217	1.002 (0.737-1.360)
Rural	2.021 (1.686-2.746)	1.700 (1.317-2.194)	2.152 (1.686-2.746)	1.680 (1.210-2.094)	0.974 (0.781-1.214)	1.062 (0.842-1.339)	0.898 (0.723-1.116)	1.006 (0.837-1.208)

^a^COR: Unadjusted odds ratios.

^b^AOR: Adjusted for Age, Division, Residency, Education, Religion, Wealth index, Employment, Newspaper, TV or radio use.

## Discussion

### Principal Findings

In this study, we aimed to measure the prevalence of mobile phone use for health care services among adult women in Bangladesh and the association between its use and uptake of essential maternal health care services and postnatal care for newborns. Our findings indicate that little higher than a quarter of the respondents reported using mobile phones to get health services. Important sociodemographic variations were observed in the use of mobile phones. The percentage was noticeably higher among those aged between 20 and 29 years, those who were from Dhaka and Chittagong division, those who lived in rural areas, and those who had primary-secondary level education. The reason behind this could be that women of this age bracket are more likely to use mobile and internet technologies, be concerned about reproductive health, and take proactive measures to ensure better pregnancy outcomes. Presumably, the higher percentage in the rural areas can be indicative of lower concentration of health facilities or lower provider to patient ratio and consequently higher dependency on distant consultations over mobile phones. Even those with no educational attainment reported using mobile phones for seeking health care advices. Another interesting finding was that the rate of using mobile phones increased linearly with the wealth quintile of the households with the rate being lowest in poorest and highest among the wealthiest households, indicating the mediating role of financial well-being in the association. Mobile phone use may also play a strong, enabling role among women lacking access to urban facilities. As the findings further suggested, the odds of having 3+ ANC visits and health facility delivery in relation to mobile phone use were higher among rural women than urban women. Women who were employed had lower rates of mobile phone use for health care seeking purposes, perhaps because they are more empowered or likely to make direct contacts with health centers. Users of electronic and print media (TV, radio, and newspaper) were also more likely to use mobile phones for seeking health care. This relationship is an intuitive one as print and electronic media have become important tools for health communication especially in the areas of maternal and child health.

Bivariate analysis revealed that those who reported ever using mobile phones to get health services had significantly higher rates of attending the minimum recommended number of ANC visits, as well as delivering at a health facility, but not for postnatal checkup among mothers and babies. These findings were reaffirmed by multivariable analysis adjusted for potential confounding variables. The odds of having 3+ ANC visits and delivering at a health facility were significantly higher among the users of mobile phones for health care purposes. These associations were further investigated by stratifying for urban and rural areas. It appeared that the odds of availing ANC and facility delivery services were higher among rural residents than urban residents.

### Past Studies and Future Research Directions

Mobile-based health service delivery strategies have attracted considerable attention as a means of supporting maternal, neonatal, and child health in developing countries [15,22,23]. There is a growing body of literature documenting the effectiveness of mHealth in improving the utilization of MHSs. However, there remains a dearth of observational and cohort studies on the effectiveness of mHealth in promoting MHSs in Bangladesh. Similar to the findings of this study, in a previous research, we have shown the disparities in the use of mobile phones for seeking childbirth services in urban Bangladesh and found that women using mobile phones for health care were more likely to deliver at a health facility [1]. A recent trial study in Ethiopia reported a positive contribution of SMS text message-based mobile phone intervention in most of the selected maternal and child health service indicators, such as improvement in the percentage of the recommended number of ANC visits, percentage of deliveries attended by health workers, and facilitating the work processes of the health workers in rural areas [24]. Another trial study made similar conclusions based on the evidence on the effectiveness of voice messages for the early initiation of MHSs [25]. However, to date, there is no evidence on the effectiveness of mHealth in promoting all the components of maternal and neonatal care. Future studies on mHealth within the context of maternal health care in Bangladesh should focus on a broader category of services and the impact of SMS text messaging services in promoting maternal health literacy and utilization of essential MHSs.

### General Discussion

Despite increasing local and international commitments to reducing maternal and child mortality rates, each year, a remarkably high percentage of women are dying owing to pregnancy and childbirth complications, a large proportion of which could have been avoided through providing essential MHSs [26-28]. In low-income countries such as Bangladesh where health care systems are fraught with infrastructure and human resource crises, the application of mHealth technologies remains a huge untapped resource. Health care experts in Bangladesh are beginning to realize the potential of health technologies and are developing policies leveraging the widespread penetration of telecommunication market to address key public health issues especially in the areas of family planning, sexual, and reproductive health care. However, shifting from traditional mode of care delivery and management to technology-driven environments will require an investment in training telemedicine facilitators and a supply of skilled professionals [29-31]. One review article mentioned that limited internet bandwidth, high cost of infrastructure, and software development are some of the main barriers to the adoption of telemedicine in Bangladesh [17]. For capacity building in this sector, university and community clinic-based telemedicine training programs for providers and community dwellers can prove highly beneficial. Finally, reaping the benefits of these programs to promote health care especially among the marginalized population will require addressing the digital divide by making sure that people have physical access and the necessary skills to properly utilize the technologies [32,33].

### Strengths and Limitations

As far as we are concerned, this has been the very first study to report the sociodemographic pattern of mobile phone use for health services among women and its relationship with the uptake of essential MHSs in any developing country. The survey was representative of the country because the sample population was selected from all the districts in the country. Given the low uptake of maternal health care services and expansion of the telecommunications sector in Bangladesh, studies of this kind are crucial to designing mHealth interventions for maternal and reproductive health.

Among the limitations was the cross-sectional and secondary nature of the data. There was no detailed information regarding the subjective report on the benefits of using mobile phone for service uptake. It is also possible that women using mobile phones were more aware of reproductive health and were socioeconomically more empowered, which are strong determinants of using health care services. There is also no indication regarding the quality of the services and whether the proportion of women who use mobile phones for health care purposes experience better reproductive outcomes. Variables were self-reported and therefore remain subject to reporting or recall error. Because the information on outcome and explanatory variables were collected at the same time, our analysis cannot suggest any causal relationship between the use of mobile phones and uptake of maternal health care services. Despite the limitations, this study offers several important insights for maternal health care programs in Bangladesh and calls for further research to investigate the quality of pregnancy outcomes among mobile phone users.

### Conclusion

Based on the analysis of BDHS (2014), this study found that, currently, little higher than a quarter of women aged 15-39 years are using mobile phones to get health services. However, there are important sociodemographic patters in the use of mobile phones. The findings conclude that women who are using mobile phones are more likely to use antenatal and professional delivery services than those who do not. Currently, there is not enough evidence to confirm any strong connection between mobile phone use and uptake of maternal health care services. More studies are necessary to replicate these findings. Future studies should focus on measuring the potential of mHealth technologies in meeting national maternal health care objectives and health priorities of the population.

## Abbreviations

AOR: adjusted odds ratio
ANC: antenatal care
BDHS: Bangladesh Demographic and Health Survey
EA: enumeration area
eHealth: electronic health
MDG 5: Millennium Development Goal 5
mHealth: mobile health
MHS: maternal health service
MoHFW: Ministry of Health and Family Welfare
SMS: short message service
